# Satisfaction and attitudes towards online continuous medical education and its impact on clinical practice among physiotherapists

**DOI:** 10.1186/s12909-024-05049-2

**Published:** 2024-01-17

**Authors:** Kholood Matouq Shalabi, Muneera Mohammed Almurdi

**Affiliations:** 1 Rehabilitation Sciences Department, College of Health and Rehabilitation Sciences, Princess Nourah bint Abdulrahman University, P.O.Box 84428, Riyadh 11671, Kingdom of Saudi Arabia; 2 Rehabilitation Health Sciences Department, College of Applied Medical Sciences, King Saud University, Riyadh, Kingdom of Saudi Arabia

**Keywords:** Physical therapy, Online medical education, Satisfaction, Attitude, Clinical practice

## Abstract

**Background:**

The traditional face-to-face of medical education is gradually being replaced with online education. However, the rate of adoption of online continuing medical education (OCME) as a learning method among practicing clinical physiotherapists (PTs) is unclear. The objectives of this study were to measure the satisfaction with, attitudes towards, and impact of OCME among practicing clinical PTs in Saudi Arabia (SA) and to examine the factors that affect the findings for satisfaction, attitude, and impact towards OCME.

**Methods:**

This cross-sectional survey was conducted between October 2021 and January 2022. PTs employed at various medical facilities and specialties in Saudi Arabia completed an online survey to assess satisfaction with, attitudes towards, and impact of OCME.

**Results:**

Of the 127 participants, 48 were female (37.8%), 44.1% were aged between 24 and 30 years. Overall, 57.5% of the respondents were satisfied with OCME compared with conventional face-to-face education, and 45.7% agreed and 18.1% strongly agreed that OCME was more flexible. Further, 52.8% of the respondents thought that OCME programs could supplement traditional face-to-face education. The majority of the participants (63.8%) agreed that participating in OCME programs increased their knowledge, and 55.1% and 51.2% agreed that attending these programs improved patient outcomes and increased their confidence in patient management, respectively. However, only 38.6% agreed that participating in OCME programs enhanced their clinical expertise. The mean satisfaction, attitude, and impact scores differed significantly according to age group, marital status, number of years of practice, and specialty (*p* < 0.0001). Multiple regression analysis showed that older age was independently associated with better satisfaction and more positive attitudes and impact. Further, having a specialization also seemed to improve the impact of OCME.

**Conclusion:**

The PTs were satisfied with and had positive attitudes towards OCME, and also found that it had a positive impact on their clinical practice. Thus, existing OCME programs are a good option for expanding the number of PTs proficient in clinical care.

**Supplementary Information:**

The online version contains supplementary material available at 10.1186/s12909-024-05049-2.

## Background

Medical education has traditionally used a conventional face-to-face learning approach [1]. With the development of technologies, education has shifted away from the conventional teacher-centered face-to-face model to online courses [2–4]. Online education is defined as teaching courses that are delivered using electronic technology and media [5]. This model provides easier and more flexible access to a huge amount of information [6]. Although the online educational approach initially met some resistance in the medical field, it has since been implemented across different medical specialties [4], including physiotherapy.

Physiotherapists (PTs) play an essential role in the delivery of quality healthcare and help individuals reach their full potential through a focus on human function and mobility [7], especially in the field of chronic illnesses [8]. Becoming a PT requires a high level of training, experience, and skill [9]. The education of PTs is vital not only at the college education level but also at the postgraduate level, because physiotherapy is an evolving and rapidly changing discipline that needs to adapt to medical advances and new information. Further, providing care for patients with chronic conditions requires patient engagement and empowerment as well as continuous improvement in the knowledge and skills of PTs in both the short and long-term. Thus, PTs opt for continuing education programs to improve and update their knowledge and skills. Continuing medical education (CME) is a key component of good clinical practice and incorporating medical advances into practice. All physicians, especially those who are not recent graduates, must therefore have access to CME to maintain lifelong continuous medical learning and provide excellent medical practice that is up to date with the latest advances in their field [10, 11]. Most medical educators acknowledge the importance of CME for individuals, academic institutions, and professional associations [12], and official CME requirements and programs represent the main means for clinicians to maintain their medical knowledge [13]. The aims of these programs are to preserve, enhance, and promote the medical professionals’ provision of healthcare [14]. However, CME cannot be maintained solely through conventional means, especially with the rapid advances occurring in all medical specialties [4]. Internet technology has helped immensely in the development and dissemination of such programs, and many CME programs have now been transformed into online continuing medical education (OCME) courses and are being implemented.

Although PTs regularly participate in OCME programs to enhance their general clinical performance and improve patient outcomes [15], transitioning from conventional to online learning is not without its barriers and challenges [16]. The success of an online learning program is determined by many student- and staff-led factors [17]. The key barriers to online learning include poor clinical skills training, a lack of institutional strategies, a lack of support, time constraints, a less personalized learning experience, and negative physicians’ attitudes towards OCME [11]. Despite these hurdles, OCME has gradually gained acceptance, especially among postgraduates [18, 19]. Further, its acceptance has been accelerated by the COVID-19 pandemic [20]. A study conducted in Turkey on the acceptance and attitudes of distance education during the COVID-19 pandemic among undergraduate PTs and rehabilitation students reported acceptance of this mode of education and a positive attitude towards it [21]. Further, another study from Singapore confirmed the general satisfaction with online education during COVID-19 among medical university students [22]. However, a study done in Croatia that examined the effect of the complete shift from face-to-face education to electronic learning (e-learning) during COVID-19 pandemic on undergraduate university health sciences students found that while most students were satisfied with the shift, a few students were worried about the lack of practical sessions [23]. Similar to other countries, medical education and healthcare organizations were significantly impacted by the COVID-19 pandemic in Saudi Arabia, and several online learning management systems were employed throughout the pandemic to assist in achieving the learning objectives [24]. As reported in other studies, a qualitative study done on 60 medical students in Saudi Arabia also found that there were some challenges to the implementation of online education systems, such as technical problems related to internet use and online exams [25].

With regard to PTs, only one other study from Saudi Arabia has examined physiotherapists’ attitudes towards and satisfaction with a webinar-based teaching learning program on chronic lower back pain [26]. Although OCME programs have now been implemented in several medical specialties in Saudi Arabia [27–30], to the best of our knowledge, no study has yet evaluated PTs’ satisfaction with and attitudes towards OCME and the impact of OCME on clinical practice in Saudi Arabia. Such as study would enable us to understand whether OCME is well accepted among PTs and what roles OCME for PTs could play in the future in the multidisciplinary care of patients. Moreover, their attitudes towards these initiatives could positively affect clinical practice and improve their patients’ outcomes. Therefore, the aims of this study were to assess PTs’ satisfaction with and attitudes towards OCME and its impact on their clinical practice. And to examine the factors that affect the findings for satisfaction, attitude, and impact towards OCME. We sought to answer the following research questions:

1. Are PTs with expertise in clinical practice satisfied with OCME and do they have positive attitudes towards OCME?
2. Does OCME impact PTs’ medical knowledge and clinical practice and improve their patients’ outcomes?
3. What are the factors that affect satisfaction with and attitudes towards OCME and its impact on clinical practice?

## Materials and methods

### Study design

This cross-sectional study included PTs working in different medical facilities and specialties in Saudi Arabia and was conducted between October 2021 and January 2022.

### Setting

Rehabilitation Sciences Department, College of Health and Rehabilitation Sciences, Prince Nourah bint Abdulrahman University, Riyadh, Kingdom of Saudi Arabia.

### Sample size calculation

The sample size was calculated using an online sample size calculator (https://www.calculator.net/sample-size-calculator.html). Assuming a 75% level of satisfaction towards OCME, with a precision of 7% and at a level of significance of 0.05, the required sample size was 147.

### Sampling technique

A consecutive, non-random sampling method was used. All Saudi Arabia-based PTs of both sexes and of all ages currently or previously enrolled in any OCME program in their area of expertise were included.

### Data collection tool

Data were collected using a questionnaire-based survey that was divided into four sections. The first section gathered demographic information about the participants, including age, gender, nationality, residency, marital status, highest medical degree, number of years of PT practice (work experience), number of months they had been enrolled in an OCME program, and their computer skill level. The second, third, and fourth sections measured the participants’ attitudes toward OCME programs, their satisfaction with the OCME programs, and the impact of these programs on their clinical practice, respectively. Participant satisfaction was assessed via nine questions pertaining to their satisfaction with the OCME programs’ overall quality, time, schedule flexibility, content, interactions, tutor support, answering of questions, practical utility, and overall satisfaction. The participants’ attitudes towards OCME were assessed via seven questions related to their perceptions about the flexibility and difficulty of these programs compared to traditional CME programs, the possibility that these OCME programs might fully or partially replace face-to-face traditional CME programs, whether they wished to be enrolled in similar OCME programs, whether they would recommend these programs to their colleagues, and whether they preferred face-to-face programs. The impact of the OCME program on their clinical practice was assessed via four questions about their perceptions about the extent of contribution of the program they are/were enrolled for in improving their medical knowledge, clinical skills, patient outcomes, and their confidence in managing patients. For statements related to attitudes and impact of the OCME programs, participants were asked to indicate their level of agreement on a Likert scale of 1 to 5 (“strongly disagree,” “strongly agree,” “neither agree nor disagree,” “disagree,” or “agree”). For the statements related to satisfaction, they also indicated their response on a Likert scale of 1 to 5 (“strongly dissatisfied,” “strongly satisfied,” “neither satisfied nor dissatisfied,” “dissatisfied,” or “strongly dissatisfied”).

### Data collection method

After obtaining their written consent the administrators of the various OCME courses provided us with their WhatsApp or email addresses. All PTs fulfilling the inclusion criteria were invited to participate in the study, and a link to the survey (created using Google Forms) was distributed to participants via email and WhatsApp. Prior to data collection, the validity of the items in the scale was assessed by calculating the content validity.

### Content validity

Content validity was assessed by a panel of 10 experts, who used the face-to-face method to calculate the content validity index (CVI) for item (I-CVI) and scale (S-CVI). S-CVI was calculated based on the average I-CVI scores for all items on the scale (S-CVI/Ave) and the proportion of items considered relevant by all experts (S-CVI/UA). The method used is that described by Yusoff (2019), according to which an acceptable CVI value for a panel of 10 experts is at least 0.78 [31].

### Statistical analysis

Data were analyzed using IBM SPSS v26.0 (IBM Corp., Armonk, N.Y., USA). Descriptive statistics (mean ± standard deviation and frequencies with percentages) were used to describe the quantitative and categorical outcome variables. Non-parametric Pearson’s chi-squared test was used to compare the ordinal scale responses for satisfaction, attitude, and impact. The five-point ordinal responses were converted into scores. Student’s *t*-test for independent samples and one-way analysis of variance (ANOVA) followed by Tukey’s multiple comparisons test were used to compare the mean satisfaction, attitude, and impact scores in relation to the sociodemographic and profession-related variables of the study participants. Multiple linear regression was used to identify the independent variables related to the scores for satisfaction, attitude, and impact. Dummy variables were created for categorical independent variables. The coefficient of variability (R-square) and regression coefficients were used to report the significance of the three models and the independent variables. A *p*-value of < 0.05 was regarded as statistically significant.

### Ethical approval

The ethical guidelines of the institutional and/or national research committees, the Helsinki statement, or comparable criteria, were followed throughout this study involving human participants. The study was planned and carried out in conformity with the ethical code of the university and with its approval (IRB approval number 21–0479).

## Results

### Participant characteristics

Out of the 147 respondents who were initially included in the survey, 20 were excluded because they did not complete the survey. Thus, the response rate was 86%. Among the 127 participants, 62.2% and 37.8% were male and female, respectively; 44.1% were aged between 24 and 30 years; 55.9% were married; and 84.3% were natives of Saudi Arabia. Most participants had a bachelor’s degree in physiotherapy (42.5%), while 29.9% had a master’s degree, 12.6% had a diploma or DPT (12.6%), and 15% had a Ph.D. The participants represented several specialties: musculoskeletal disorders (31.5%), general physiotherapy (22.8%), neurology (13.4%), pediatrics (11.8%), and sport (15.0%). With regard to experience, 23.6% had 0 to 2 years of experience; 22.8%, over 15 years; 20.5%, 6 to 10 years; 19.7%, 3 to 5 years; and 13.4%, 11 to 15 years (Table 1).

**Table 1:** Socio-demographic and professional characteristics of the study subjects (*N* = 127)

Variable	Characteristics	*N* (%)
Gender	Male	79 (62.2)
	Female	48 (37.8)
Age group (years)	25–29	56 (44.1)
	30–34	22 (17.3)
	35–39	18 (14.2)
	>=40	31 (24.4)
Marital Status	Single	56 (44.1)
	Married	71 (55.9)
Nationality	Saudi	107 (84.3)
	Non-Saudi	20 (15.7)
Academic degree	Ph.D	19 (15)
	Master’s	38 (29.9)
	BPT	54 (12.5)
	Diploma and DPT	16 (7.9)
Specialty	Sport	15 (11.8)
	Pediatric	40 (31.5)
	Neurology	17 (13.4)
	Musculoskeletal	40 (31.5)
	General	29 (22.8)
	Others	7 (5.5)
PT practice (years)	0–2	30 (23.6)
	3–5	25 (19.7)
	6–10	26 (20.5)
	11–15	17 (13.4)
	> 15	29 (22.8)

BPT: Bachelor Physical Therapy, DPT: Doctorate Physical Therapy

### Content validity

I-CVI, S-CVI/Ave, and S-CVI/UA were above the minimum cut-off of 0.78, and their values confirmed the validity of the tool. With regard to I-CVI, the value was 1 for 19 questions; this indicates 100% agreement among the 10 experts. The remaining question had a CVI of 0.8, which was still above the cut-off of 0.78. The S-CVI/Ave and S-CVI/UA were 0.99 and 0.95, respectively. These values indicate the high content validity of the tool.

### Satisfaction with OCME

The majority of the respondents (over 50%) reported that they were either “strongly satisfied” or “satisfied” with 8 out of the 9 items in this section, namely, quality, time spent, schedule flexibility, interactions during the program, content, tutor support, answering of questions, and overall satisfaction. However, a smaller percentage, that is, 46.5%, reported that they were “strongly satisfied” or “satisfied” with the practical usefulness of the program. This indicates that the PTs were largely satisfied with their OCME program experiences (Table 2).

**Table 2:** Distribution and comparison of satisfaction item responses about online continuous medical education (OCME).

Items	Responses				
	Strongly satisfied *N* (%)	Satisfied *N* (%)	Neither satisfied nor dissatisfied *N* (%)	Dissatisfied *N* (%)	Strongly dissatisfied *N* (%)
To which degree are you satisfied with the overall quality of the OCME program/s you are/were enrolled in?	14 (11.0)	67 (52.8)	27 (21.3)	15 (11.8)	4 (3.1)
To which degree are you satisfied with the time you spend on learning via OCME programs?	8 (6.3)	67 (52.8)	33 (26.0)	11 (8.7)	8 (6.3)
To which degree are you satisfied with the schedule flexibility of the OCME programs?	20 (15.7)	58 (45.7)	35 (27.6)	10 (7.9)	4 (3.1)
To which degree are you satisfied by the interactions you have during OCME programs?	12 (9.4)	59 (46.5)	34 (26.8)	15 (11.8)	7 (5.5)
To which degree are you satisfied with the content provided by the OCME programs?	17 (13.4)	69 (54.3)	34 (26.8)	4 (3.1)	3 (2.4)
To which degree are you satisfied with the tutor support provided during the OCME programs?	9 (7.1)	66 (52.0)	37 (29.1)	8 (6.3)	7 (5.5)
To which degree are you satisfied with your question answering during the OCME programs?	12 (9.4)	62 (48.8)	35 (27.6)	13 (10.2)	5 (3.9)
To which degree are you satisfied with the practical usefulness of the OCME programs?	9 (7.1)	50 (39.4)	36 (28.3)	23 (18.1)	9 (7.1)
How do you rate your overall satisfaction with the OCME (or you have attended)?	8 (6.3)	73 (57.5)	33 (26.0)	9 (7.1)	4 (3.1)

### Attitude towards OCME

For four of the items in the attitudes section, as listed below, > 60% of the PTs reported that they “strongly agreed” or “agreed:” “online CME programs are more flexible than traditional face-to-face education,” “online CME programs can partially replace traditional face-to-face education in some aspects,” “I would enroll in other online CME programs if there would be any in the future,” and “I would recommend my colleagues to enroll in online CME programs.” In addition, a majority (54.3%) of the participants also reported that they “strongly agreed” or “agreed” with the statement “I prefer face-to-face CME programs such as conferences and seminars to online CME programs.” With regard to the remaining two items (“online CME programs are more difficult than traditional face-to-face education” and “online CME programs can fully replace traditional face-to-face education”), only 34.6% and 31.5% of subjects reported that they “strongly agreed” and “agreed.” Overall, the PTs had a positive attitude for five out of the seven items in the section on attitudes towards OCME (Table 3).

**Table 3:** Distribution and comparison of attitude items towards online continuous medical education (OCME).

Items	Responses				
	Strongly agree *N* (%)	Agree *N* (%)	Neither agree nor disagree *N* (%)	Disagree *N* (%)	Strongly disagree *N* (%)
To which degree do you agree with this statement: Online CME programs are more flexible than traditional face-to-face education	23 (18.1)	58 (45.7)	19 (15)	17 (13.4)	10 (7.9)
To which degree do you agree with this statement: Online CME programs are more difficult than traditional face-to-face education.	8 (6.3)	36 (28.3)	34 (26.8)	38 (29.9)	11 (8.7)
To which degree do you agree with this statement: Online CME programs can fully replace traditional face-to-face education.	9 (7.1)	31 (24.4)	36 (28.3)	30 (23.6)	21 (16.5)
To which degree do you agree with this statement: Online CME programs can partially replace traditional face-to-face education in some aspects	16 (12.6)	67 (52.8)	30 (23.6)	9 (7.1)	5 (3.9)
To which degree do you agree with this statement: I would enroll in other online CME programs if there would be any in the future	22 (17.3)	68 (53.5)	26 (20.5)	5 (3.9)	6 (4.7)
To which degree do you agree with this statement: I would recommend my colleagues to enroll in online CME programs	22 (17.3)	66 (52.0)	25 (19.7)	8 (6.3)	6 (4.7)
To which degree do you agree with this statement: I prefer face-to-face CME programs such as conferences and seminars to online CME programs.	15 (11.8)	54 (42.5)	39 (30.7)	15 (11.8)	4 (3.1)

### Impact of OCME on the participants’ clinical practice

Over 70% of the PTs “strongly agreed” and “agreed” with the statement “attending an OCME program improved my medical knowledge,” while 62.2% and 59.1% “strongly agreed” and “agreed“ with the statements “attending an OCME program improved my patient outcomes” and “after attending an OCME program, I feel confident to manage my patients,” respectively. With regard to the remaining item (“attending an OCME program improved my clinical skills”), only 46.5% reported that they “strongly agreed” and “agreed.” These responses indicate that the participants felt that their practice was mostly positively impacted by OCME (Table 4).

**Table 4:** Distribution and comparison of impact items responses towards online continuous medical education (OCME).

Items	Responses				
	Strongly agree *N* (%)	Agree *N* (%)	Neither agree nor disagree *N* (%)	Disagree *N* (%)	Strongly disagree *N* (%)
To which degree do you agree with this statement: Attending an OCME program improved my medical knowledge	17 (13.4)	81 (63.8)	23 (18.1)	2 (1.6)	4 (3.1)
To which degree do you agree with this statement: Attending an OCME program improved my clinical skills.	10 (7.9)	49 (38.6)	41 (32.3)	20 (15.7)	7 (5.5)
To which degree do you agree with this statement: Attending an OCME program improved my patient outcomes.	9 (7.1)	70 (55.1)	32 (25.2)	10 (7.9)	6 (4.7)
To which degree do you agree with this statement: after attending an OCME program, I feel confident to manage my patients.	10 (7.9)	65 (51.2)	36 (28.3)	11 (8.7)	5 (3.9)

### Association of satisfaction, attitude, and impact scores with sociodemographic and profession-related variables

The mean satisfaction, attitudes, and impact scores significantly differed according to participant age (*p* < 0.0001, *p* = 0.001, and *p* = 0.020, respectively). That is, the mean values of these scores were significantly higher in the age groups 36–40 years and > 40 years than in the age groups 25–29 and 30–34 years. The mean attitude and impact scores also significantly differed according to marital status (*p* = 0.022 and *p* = 0.017 for differences in the attitude and impact scores, respectively), with married respondents having higher mean attitude and impact scores than those who were single. The mean impact scores were significantly higher in PTs specializing in neurology, musculoskeletal, and other specialties (*p* = 0.010). Moreover, the impact scores were higher for those who had practiced PT for longer: that is, they were higher for those with 11–15 and > 15 years of experience than in those who only had 0–2, 3–5, or 6–10 years of experience (*p* = 0.006). The mean values of these three scores did not significantly differ with respect to gender, nationality, or academic degree (Table 5).

**Table 5:** Comparison of mean values of satisfaction, attitude, and impact scores towards OCME with respect to the socio-demographic and professional characteristics of study subjects

**Characteristics**	**Satisfaction scores**			Attitude scores			Impact scores		
	Mean (SD)	*F*-value/*t*-value	*p*-value	Mean (SD)	*F*-value/*t*-value	*p*-value	Mean (SD)	*F*-value /*t*-value	*p*-value
Age groups									
25–29	30.5 (6.2)	9.92	< 0.0001*	23.5 (4.4)	5.78	0.001*	13.8 (2.7)	3.38	0.020*
30–34	33.5 (3.5)			24.9 (4.5)			13.4 (1.7)		
35–39	36.1 (3.4)			26.8 (2.4)			14.4 (3.2)		
>=40	35.5 (3.6)			26.7 (3.3)			15.4 (2.9)		
Gender									
Male	32.8 (5.7)	-0.75	0.452	24.7 (4.6)	-1.18	0.241	14.9 (2.6)	1.82	0.071
Female	33.5 (4.9)			25.6 (3.3)			14.0 (2.9)		
Marital status									
Single	32.4 (5.8)	-1.26	0.210	24.1 (5.0)	-2.32	0.022*	13.9 (3.2)	-2.42	0.017*
Married	33.6 (5.1)			25.8 (3.2)			15.1 (2.2)		
Nationality									
Saudi	33.0 (5.7)	-0.44	0.658	24.9 (4.4)	-0.85	0.399	14.5 (2.9)	-0.63	0.529
Non-Saudi	33.5 (3.6)			25.7 (2.7)			14.9 (1.8)		
Academic degree									
Ph.D.,	34.6 (4.7)	0.92	0.433	25.3 (3.3)	1.92	0.130	15.3 (2.5)	1.61	0.191
Masters	33.2 (4.5)			25.2 (3.2)			14.6 (1.9)		
Diploma & DPT	33.4 (6.2)			26.9 (4.4)			13.3 (3.8)		
BPT	32.3 (5.9)			24.2 (4.8)			14.6 (2.9)		
Specialty									
Sport	34.0 (4.7)	0.96	0.445	25.7 (4.9)	2.03	0.079	13.5 (2.9)	3.17	0.010*
Pediatric	32.2 (5.2)			24.3 (2.8)			13.8 (2.2)		
Neurology	31.2 (5.3)			25.6 (2.9)			14.8 (2.4)		
Musculoskeletal	33.9 (4.5)			25.7 (3.1)			15.5 (2.3)		
General	32.4 (7.2)			23.4 (5.6)			13.7 (3.3)		
Others	34.6 (3.6)			26.9 (3.9)			16.4 (1.4)		
PT practice (years)									
0–2	31.9 (6.6)	3.82	0.006*	23.7 (5.1)	1.33	0.262	13.5 (3.5)	1.62	0.172
3–5	31.6 (5.0)			25.9 (3.6)			15.1 (2.6)		
6–10	30.2 (6.6)			24.6 (5.3)			14.5 (2.5)		
11–15	33.5 (4.1)			25.2 (2.7)			14.5 (2.2)		
> 15	35.9 (3.2)			25.8 (2.8)			15.1 (2.2)		

*Statistically significant at the *p* < 0.05 level

### Multiple linear regression analysis

In order to identify the factors that were independently associated with the satisfaction, attitude, and impact scores, multiple linear regression models were generated with satisfaction scores, attitude scores, and impact scores as dependent variables and the following bivariate significant variables as independent variables: age groups (25–29, 30–34, 35–39, and ≥ 40 years); marital status (single and married); specialty (sports, pediatrics, neurology, musculoskeletal disorders, general physiotherapy, and others); and years of experience (0–2, 3–5, 6–10, 11–15, and > 15 years).

In the model in which satisfaction score was the dependent variable, the age groups 30–34, 35–39, and ≥ 40 years were identified as significant, independent factors related to the satisfaction score. That is, the satisfaction scores significantly increased, on average, by 2.807, 5.923, and 4.394 for the age groups 30–34 (*p* = 0.022), 35–39 years (*p* < 0.0001), and ≥ 40 years (*p* = 0.0001), respectively, when compared with the age group 25–29 years. The model had an R-square value of 0.266, which indicates that 26.6% of the difference in satisfaction scores was explained by the three age groups. Finally, the model was found to be statistically significant (F = 6.165, *p* < 0.0001) (Table 6).

**Table 6:** Multiple linear regression analysis for relationship between (i) Satisfaction score (ii) Attitude scores (iii) Impact scores and independent variables

Outcome variable	Independent Variables	Unstandardized Coefficients		Standardized Coefficients	*t*-value	*p*-value	95.0% Confidence Interval for B	
		B	Std. Error	Beta			Lower Bound	Upper Bound
Satisfaction Score	(Constant)	30.049	0.974	-	30.855	< 0.0001	28.12	31.98
	Age group (30to 34)	2.807	1.213	0.197	2.315	0.022	0.41	5.21
	Age group (35 to 39)	5.923	1.342	0.383	4.413	< 0.0001	3.26	8.58
	Age group > = 40	4.394	1.204	0.350	3.649	< 0.0001	2.01	6.78
Attitude Score	(Constant)	23.057	0.611	-	37.751	< 0.0001	21.85	24.27
	Age groups (35 to39)	3.038	1.074	0.255	2.828	0.005	0.91	5.16
	Age group > = 40	2.892	0.904	0.299	3.199	0.002	1.10	4.68
Impact Score	(Constant)	12.669	0.682	-	18.582	< 0.0001	11.32	14.02
	Age group > = 40	1.309	0.601	0.206	2.177	0.031	0.12	2.50
	Specialty (Musculoskeletal)	1.890	0.732	0.321	2.583	0.011	0.44	3.34
	Specialty (Others)	2.426	1.182	0.203	2.053	0.042	0.08	4.77

The second model with attitude score as a dependent variable showed that the age groups 35–39 and ≥ 40 years were significantly associated with the attitude scores. That is, the attitude scores significantly increased, on average, by 3.038 and 2.892 in the age groups 35–39 (*p* = 0.005) and ≥ 40 years (*p* = 0.002), respectively, when compared with the age group 25–29 years. The model shows an R-square value of 0.140, which indicates that 14.0% of the difference in the attitude scores was explained by the two age groups. Finally, the model was found to be statistically significant (F = 4.968, *p* = 0.001) (Table 6).

The third model with impact score as the dependent variable showed that the age group ≥ 40 years and the specialties musculoskeletal and others were significantly related to the impact scores. The regression coefficients indicate that the impact scores, on average, significantly increased by 1.309 in the age group ≥ 40 years when compared with the age group 25–29 years (*p* = 0.031). Further, it significantly increased by 1.890 and 2.426 for the specialty musculoskeletal disorders (*p* = 0.011) and others (*p* = 0.042), respectively, when compared with the specialty sports. The model shows an R-square value of 0.179, which indicates that 17.9% of the change in impact scores was explained by the age group ≥ 40 years and the two types of specialties. Moreover, this model was also statistically significant (F = 2.826, *p* = 0.005) (Table 6).

Thus, older age was associated with significantly better satisfaction, attitude, and impact scores, and impact scores were also affected by the specialty of the PT.

## Discussion

This study aimed to assess the satisfaction with and attitudes towards OCME programs among PTs and their perception of the impact of the programs on their clinical practice. In addition, the factors that affected the satisfaction, attitudes, and impact were also analyzed. The results indicated that the PTs were largely satisfied with their OCME programs and had a positive attitude towards it, and the impact was mostly considered to be positive. With regard to the influential factors, older participants reported higher satisfaction, more positive attitudes, and more positive impact. Moreover, those with a specialization in musculoskeletal disorders and other areas also reported a more positive impact of the programs on their clinical practice.

Most of our respondents were Saudi nationals (84.3%). This highlights the growing local interest in PT and the rising number of students enrolling in PT programs in Saudi Arabia. In addition, over the last 10 years, the number of universities offering a bachelor’s degree in PT has increased from 6 to 16 universities (14 public and 2 private) [32]. This upturn in interest and engagement might partly be due to the Saudi Vision 2030 initiatives and the growing healthcare demands in Saudi Arabia. Given the increasing implementation of online learning systems, this trend points to the need for much more research on how students and healthcare professionals perceive these systems and how the online learning and training systems can be optimized to meet their needs and, subsequently, improve clinical practice and patient outcomes.

With regard to assessment of satisfaction in the present study, the current participants were mostly satisfied with eight of the nine items (quality, time, schedule flexibility, tutor support, answering of questions, interactions, content, and overall satisfaction). In particular, 63.8% of the PTs stated that they were “strongly satisfied” and “satisfied” in terms of overall satisfaction with the OCME programs, while 26% were neither satisfied nor dissatisfied. Thus, overall, the PTs appeared to be satisfied with the OCME programs over the study period. These results are consistent with those of another study in Saudi Arabia conducted by Nambi et al., [26] who found that PTs trained through webinars were satisfied with their training programs. In addition, a study on an e-learning program for fall prevention for PTs reported that the participants were satisfied with the program overall and found it acceptable, and another study on online training for PTs (through video conferencing) reported that they gave the training a moderate-to-high rating for effectiveness and expressed satisfaction with it [33]. Similarly, a study conducted in South Korea on physicians engaged in CME activities showed that 85.21% of the participants were satisfied with the online education activities [34]. The positive response to the flexibility of the online program observed in the present survey is echoed in a study on hematology/oncology fellows who received online medical education during the COVID-19 pandemic, as 100% reported that they appreciated the flexibility of the online learning environment [35]. In contrast to these findings, a Chinese (preprint) study on a full online teaching program conducted on 61 physical therapy students during the final phase of the pandemic showed that satisfaction with teaching strategies dropped significantly [36]. In addition, it has been reported that PT students felt that online education during periods of emergency (i.e., during the COVID-19 pandemic) was suboptimal for both the theoretical and practical course components, and they were unsatisfied with it [37]. Especially in universities or colleges, PT students may feel that hands-on teaching is more appropriate for learning practical treatment techniques and skills. In the case of our participants, most PTs had at least 2 years of work experience, so they probably already had a better understanding of the treatments.

In terms of attitudes, 45.7% of our cohort “agreed” and 18.1% “strongly agreed” that OCME programs are more flexible than traditional face-to-face education. The other study on online education among PTs conducted in Saudi Arabia by Nambi et al. also reported that their attitudes had improved at the end of the course [26]. Similarly, a study done by Şavkın et al. (2021) [21] in Turkey during the COVID-19 pandemic found that the attitudes towards distance learning among PTs and rehabilitation undergraduate students were positive. The study also found that acceptance was higher among first and fourth academic year students than among second- and third-year students [21]. This is probably because the first year mainly covered theoretical courses, while all the practical courses had been completed by the fourth year. In contrast, second- and third-year students require more hands-on training and face-to-face teaching as they have more practical courses [21]. In another study, online learning was largely perceived as a flexible and convenient mode of learning, but at the same time, the majority of students (79%) thought that the online learning environment was detrimental to their comprehension of the subject and disadvantaged them compared with traditional face-to-face teaching methods [38]. Further, surveys conducted in Pakistan [39], India [40], the Philippines [41], and Poland [42] during the COVID-19 pandemic revealed that the majority of medical students had a negative opinion of or voiced discontent with online learning.

Moreover, conclusions from 2721 medical students from 39 medical schools in the UK revealed that online learning was not as effective as face-to-face learning and that there were few opportunities for students to raise questions [43]. Besides, the Zhang et al. study from China also reported that the overall trend in physical therapy students’ attitude to online learning was negative [36]. In contrast to these findings, 52.8% of our respondents agreed that OCME programs could partially replace traditional face-to-face education. As the participants of our study were PTs who were practicing their professional skills in their clinics, it was probably more convenient for them to update and improve their knowledge through online courses (as they might not have had the time for face-to-face courses). Online courses and workshops may also provide them with the opportunity to engage in social activities and improve their active participation in and interactions with the community. This idea is also supported by the study published by Şavkın et al. (2021) [21]. However, the findings in the literature are inconsistent, as one study reported no differences between face-to-face and online instruction [44]. These differences in studies could be attributable to differences in study characteristics such as the study population, age group, and experience.

The majority of the participants (about 60%) in the current study felt that the OCME programs had improved their medical knowledge, patient outcomes, and confidence in managing patients, but a smaller percentage (about 45%) felt that it had improved their clinical skills. This reflects previous findings that PTs reported improvement in knowledge through online programs [33]. Similarly, knowledge evaluation after a massive online open course showed a 20% increase in the median scores of physiotherapy students and PTs, and over 80% provided a positive evaluation of the course [45]. Additional, the study from South Korea on OCME during the pandemic reported that 87.29% of the participants felt that the content had influenced their clinical practice; further, 78.07% of the respondents indicated that they had made actual changes to their clinical practice after the program [34].

In our study, around 45% of the respondents felt that the courses had helped them to improve their clinical skills. However, a study from Guangzhou concluded that online teaching may not be suitable for the development of practical skills among students [36]. Thus, a blended mode of learning that includes both online teaching and face-to-face courses for practical skills might be an optimal strategy, as reported by Puljak et al. [23]. This notion is supported by an online survey of 2961 users (including physicians, midwives, and paramedics) of a German CME platform, according to which frequent online lectures at regular intervals (77.8%) and combined face-to-face and online CME (55.9%) were favored [46]. Further, a blended mode of delivery of a physiology subject for allied health students at the university level showed that students demonstrated better grades with the blended mode of education than traditional modes of teaching [47]. In contrast to these findings, a systematic review and meta-analysis on the effectiveness of digital learning designs in physiotherapy education on 22 studies reported that blended learning and distance learning did not significantly differ and were not more effective than traditional teaching in terms of the learning outcomes of PT students [48]. As discussed previously, while online learning and a blended method of delivery may offer convenience and flexibility for physiotherapists who are already practicing, it may not be beneficial to physiotherapy students who need hands-on experience with real patients in the clinical to improve their skills.

Multiple regression analysis in the present study showed that age was associated with the results for satisfaction, attitudes, and impact, but gender, country, and level of education were not associated with the scores. Specially, we found that older PTs had better satisfaction and reported more positive attitudes and impact. Older PTs may already have experience with traditional continuing education, such as in-person conferences and seminars, and may find online education to be sufficient for their continuing education. Further, they may find the online format to be suitable to their schedules in the clinical setting. The current findings also showed that better impact was reported by PTs with a specialization. Subspecialty, training and experience may make PTs more aware of the potential benefits of OCME, such as access to specialized content and the ability to learn at their own pace. Moreover, subspecialty training and experience indicate greater professional interest and investment in their field, which may contribute to higher satisfaction and engagement with OCME. While these findings are insightful, there are not enough studies in the literature on whether age, sex, and other factors affect satisfaction with and attitudes towards OCME. With regard to the adoption of OCME, one study reported that younger physicians are more likely to adopt OCME than older physicians [49]. However, a recent scoping review on virtual CME found that most studies do no report age [50]. Further, the sex-based differences are unclear, as one study found that male physicians were more likely to use the Internet for CME than female physicians [51], while another study found that female physicians were more likely to use online CME programs than their male counterparts [49]. Thus, there is a need for more data on age- and sex-based differences in how OCME is perceived, as this could help highlight gaps in the delivery of OCME in different demographics.

This is the first study on the satisfaction, attitude, and impact of OCME among practicing PTs in Saudi Arabia, as most previous studies were conducted on PT students. These results would, therefore, be helpful for policymakers and PTs planning to enroll for OCME activities. However, this study is limited by its cross-sectional design, the small sample size, and the unequal distribution of participants in terms of sex, age, and number of years of practice. Future research would benefit from a randomized or prospective cohort study design with more individuals and from studying new digital technologies such as augmented and virtual reality [52]. Further, in the future, the incorporation of student-centered learning in OCME through Internet-based resources, such as web alerts, medical newsletters, electronic databases, podcasts, web conferences, bibliometrics, and living systematic reviews, needs to be investigated, as they have been found to benefit clinicians, medical researchers, and students [53].

In conclusion, OCME for PTs appears to be a viable solution for CME since PTs seem to find it as satisfactory and effective as face-to-face courses. Our study illustrates the value of an OCME curriculum for improving PTs’ understanding of patient management, but it also highlights its limitations in terms of providing hands-on training in the development of clinical skills.

### Lessons for practice

- Educators should continue to develop OCME courses, which are equivalent to traditional CME.
- Outreach may be important for certain subgroups of physiotherapists to encourage the use of OCME.
- Work is still required to develop OCME that directly impacts the development of clinical skills.

### Electronic supplementary material

Below is the link to the electronic supplementary material.

Supplementary Material 1

## Data Availability

Dataset available from the corresponding author at malmurdi@ksu.edu.sa.

## Abbreviations

OCME: Online continuing medical education
CME: Continuing medical education
PTs: Physiotherapists
SA: Saudi Arabia
e-learning: Electronic learning
CVI: Content validity index
I-CVI: Item-level content validity index
S-CVI: Scale content validity
S-CVI/Ave: Scale-level content validity index based on the average
S-CVI/UA: Scale-level content validity index based on the universal agreement
