# Supplementary material for: Satisfaction and attitudes towards online continuous medical education and its impact on clinical practice among physiotherapists
Source: BMC Med Educ. 2024 Jan 17;24:70. doi: 10.1186/s12909-024-05049-2 (PMC10795308; doi:10.1186/s12909-024-05049-2)
Supplement: Supplementary file 1 — Supplementary Material 1 [file 12909_2024_5049_MOESM1_ESM.docx]

**Supplementary 1.** Survey structure and questions.

This survey aims at evaluating your satisfaction and attitude towards online continuous medical education (OCME), and its impact on your clinical practice.

**Section I: Demographic data**

- **What is your age [in years]?**

□ 24-<30

□30 -<35

□35 -<40

□40 -<45

□45 -<50

□50 -<55

□55 -<60

□>60

- **What is your gender?**

□ Male

□ Female

- **What is your nationality?**

□ Saudi

□ non-Saudi

- **What best describes your city of residence?**

□ Urban

□ Rural

- **What is your marital state?**

□ Single

□ Married

□ Divorced

□ Widow/widower

- **What is the highest medical degree you have?**

□ BPT

□ DPT

□ Master’s degree

□Ph.D. degree

□ Diploma

□ Others. Please specify: ……………………………………..

- **How many years have you been in physical therapy practice?**

Please type a number

- **What is your physical therapy specialty?**
- Please specify: ……………………………………..
- **How long have you been enrolled in an Online continuous medical education (OCME) in weeks?**

Please type a number

**Section II: Satisfaction with Online continuous medical education (OCME) you are/were enrolled in.**

Please rate each of the following about the OCME module/es you are/were enrolled in:

1. To which degree are you satisfied with the overall quality of the OCME program/s you are/were enrolled in?

□ Strongly not satisfied □ Not satisfied □ Neither satisfied nor not satisfied □ Satisfied □ Strongly satisfied

1. To which degree are you satisfied with the time you spend on learning via OCME programs?

□ Strongly not satisfied □ Not satisfied □ Neither satisfied nor not satisfied □ Satisfied □ Strongly satisfied

1. To which degree are you satisfied with the schedule flexibility of the OCME programs?

□ Strongly not satisfied □ Not satisfied □ Neither satisfied nor not satisfied □ Satisfied □ Strongly satisfied

1. To which degree are you satisfied by the interactions you have during OCME programs?

□ Strongly not satisfied □ Not satisfied □ Neither satisfied nor not satisfied □ Satisfied □ Strongly satisfied

1. To which degree are you satisfied with the content provided by the OCME programs?

□ Strongly not satisfied □ Not satisfied □ Neither satisfied nor not satisfied □ Satisfied □ Strongly satisfied

1. To which degree are you satisfied with the tutor support provided during the OCME programs?

□ Strongly not satisfied □ Not satisfied □ Neither satisfied nor not satisfied □ Satisfied □ Strongly satisfied

1. To which degree are you satisfied with your question answering during the OCME programs?

□ Strongly not satisfied □ Not satisfied □ Neither satisfied nor not satisfied □ Satisfied □ Strongly satisfied

1. To which degree are you satisfied with the practical usefulness of the OCME programs?

□ Strongly not satisfied □ Not satisfied □ Neither satisfied nor not satisfied □ Satisfied □ Strongly satisfied

1. How do you rate your overall satisfaction with the OCME you (or you have attended)?

□ Strongly not satisfied □ Not satisfied □ Neither satisfied nor not satisfied □ Satisfied □ Strongly satisfied

**Section III: Attitude towards Online continuous medical education (OCME) you are/were enrolled in.**

1. To which degree do you agree with this statement: Online CME programs are more flexible than traditional face-to-face education.

□ Strongly disagree □ Disagree □ Neither agree nor disagree □ Agree □ Strongly agree

1. To which degree do you agree with this statement: Online CME programs are more difficult than traditional face-to-face education.

□ Strongly disagree □ Disagree □ Neither agree nor disagree □ Agree □ Strongly agree

1. To which degree do you agree with this statement: Online CME programs can fully replace traditional face-to-face education.

□ Strongly disagree □ Disagree □ Neither agree nor disagree □ Agree □ Strongly agree

1. To which degree do you agree with this statement: Online CME programs can partially replace traditional face-to-face education in some aspects.

□ Strongly disagree □ Disagree □ Neither agree nor disagree □ Agree □ Strongly agree

1. To which degree do you agree with this statement: I would be enrolled in other online CME programs if they will be any in the future.

□ Strongly disagree □ Disagree □ Neither agree nor disagree □ Agree □ Strongly agree

1. To which degree do you agree with this statement: I would recommend my colleagues to be enrolled in online CME programs.

□ Strongly disagree □ Disagree □ Neither agree nor disagree □ Agree □ Strongly agree

1. To which degree do you agree with this statement: I prefer face-to-face CME programs such as conferences and seminars to online CME programs.

□ Strongly disagree □ Disagree □ Neither agree nor disagree □ Agree □ Strongly agree

**Section IV: Impact of the Online continuous medical education (OCME) you are/were enrolled in on your clinical practice.**

1. To which degree do you agree with this statement: Attending an OCME program improved my medical knowledge.

□ Strongly disagree □ Disagree □ Neither agree nor disagree □ Agree □ Strongly agree

1. To which degree do you agree with this statement: Attending an OCME program improved my clinical skills.

□ Strongly disagree □ Disagree □ Neither agree nor disagree □ Agree □ Strongly agree

1. To which degree do you agree with this statement: Attending an OCME program improved my patient outcomes.

□ Strongly disagree □ Disagree □ Neither agree nor disagree □ Agree □ Strongly agree

1. To which degree do you agree with this statement: after attending an OCME program, I feel confident to manage my patients.

□ Strongly disagree □ Disagree □ Neither agree nor disagree □ Agree □ Strongly agree
